# Comparison of Accuracy of Diabetes Risk Score and Components of the Metabolic Syndrome in Assessing Risk of Incident Type 2 Diabetes in Inter99 Cohort

**DOI:** 10.1371/journal.pone.0022863

**Published:** 2011-07-29

**Authors:** Tracy B. Shafizadeh, Edward J. Moler, Janice A. Kolberg, Uyen Thao Nguyen, Torben Hansen, Torben Jorgensen, Oluf Pedersen, Knut Borch-Johnsen

**Affiliations:** 1 Tethys Bioscience, Emeryville, California, United States of America; 2 Hagedorn Research Institute, Gentofte, Denmark; 3 Research Center for Prevention and Health, Glostrup Hospital, Copenhagen, Denmark; 4 Faculty of Health Science, University of Copenhagen, Copenhagen, Denmark; 5 Institute of Biomedical Science, Faculty of Health Sciences, University of Copenhagen, Copenhagen, Denmark; 6 Faculty of Health Science, University of Aarhus, Aarhus, Denmark; 7 Novo Nordisk Foundation Center for Metabolic Research, University of Copenhagen, Copenhagen, Denmark; 8 Institute Public Health, Research Center for Quality in Health Care, University of Southern Denmark, Odense, Denmark; University of Padova, Italy

## Abstract

**Background:**

Given the increasing worldwide incidence of diabetes, methods to assess diabetes risk which would identify those at highest risk are needed. We compared two risk-stratification approaches for incident type 2 diabetes mellitus (T2DM); factors of metabolic syndrome (MetS) and a previously developed diabetes risk score, PreDx® Diabetes Risk Score (DRS). DRS assesses 5 yr risk of incident T2DM based on the measurement of 7 biomarkers in fasting blood.

**Methodology/Principal Findings:**

DRS was evaluated in baseline serum samples from 4,128 non-diabetic subjects in the Inter99 cohort (Danes aged 30–60) for whom diabetes outcomes at 5 years were known. Subjects were classified as having MetS based on the presence of at least 3 MetS risk factors in baseline clinical data. The sensitivity and false positive rate for predicting diabetes using MetS was compared to DRS. When the sensitivity was fixed to match MetS, DRS had a significantly lower false positive rate. Similarly, when the false positive rate was fixed to match MetS, DRS had a significantly higher specificity. In further analyses, subjects were classified by presence of 0–2, 3 or 4–5 risk factors with matching proportions of subjects distributed among three DRS groups. Comparison between the two risk stratification schemes, MetS risk factors and DRS, were evaluated using Net Reclassification Improvement (NRI). Comparing risk stratification by DRS to MetS factors in the total population, the NRI was 0.146 (p = 0.008) demonstrating DRS provides significantly improved stratification. Additionally, the relative risk of T2DM differed by 15 fold between the low and high DRS risk groups, but only 8-fold between the low and high risk MetS groups.

**Conclusions/Significance:**

DRS provides a more accurate assessment of risk for diabetes than MetS. This improved performance may allow clinicians to focus preventive strategies on those most in need of urgent intervention.

## Introduction

The health and economic burden of diabetes mellitus, primarily type 2 diabetes (T2DM), is large today and expected to increase significantly in the next 15–25 years. By 2030, 4.4% (about 370 million people) of the global population is expected to have diabetes [1], a disease that is expected to rank seventh in the world as the leading cause of deaths and eleventh as the leading cause of disability adjusted life years lost [2]. In the United States in 2007, approximately 7.8% of adults (i.e. about 24 million people) had diabetes, primarily type 2, while nearly 23% of people in the age group 60 years or older were diabetic [3].

Due to the morbidity, mortality and cost associated with T2DM, it will be beneficial to identify individuals who are at higher risk for developing the disease and who specifically may benefit from intensive diabetes prevention strategies [4], [5]. Impaired fasting glucose (IFG) (i.e. fasting plasma glucose between 5.6 and 6.9 mmol/L; 100–125 mg/dL) is considered to be a prediabetic state. Indeed, 9–37% of patients with IFG develop T2DM in a period of 2.5–11.5 years [6], [7], [8], [9], [10]. Yet, this measure of glucose dysregulation is an imprecise predictor as T2DM does not develop in a large number of individuals with IFG.

The presence of metabolic syndrome has also been used to identify subjects at higher risk for the development of T2DM. Metabolic syndrome (MetS) is a cluster of risk factors that are associated with increased risk for diabetes and/or cardiovascular disease [11], [12], [13], [14]. In accordance with a recent consensus definition of MetS, these risk factors include central obesity/large waist circumference, elevated blood pressure, elevated fasting serum triglycerides, reduced fasting HDL and elevated fasting plasma glucose [12]. An individual is considered to have MetS if three of the five criteria are present [12]. It is difficult to estimate the number of people with MetS as the definitions used in various parts of the world and by various medical or health organizations differ [11]. The strong association between factors of the metabolic syndrome and incident diabetes was reviewed recently [15], [16]. Depending on the criteria used to define metabolic syndrome, the average relative risk ranged from 3.5–5.2 [15]. This association was stronger for diabetes than for coronary heart disease [16].

Recently, Nichols and Moler assessed the risk of developing T2DM in a cohort of 58,056 North American subjects ≥30 years old based on whether or not levels of the five MetS components were abnormal at baseline [17]. Risk of T2DM increased with the number of abnormal MetS components present. However, the incidence of T2DM associated with three or more MetS factors ranged between 1.8% and 28.2% depending on which specific combination of factors was present. This variability of risk associated with solely counting the number of abnormal MetS components may limit their use for assessment of risk for T2DM in clinical practice.

Another approach for assessing risk of T2DM utilizes risk scores or models [18], [19], [20], [21], [22], [23], [24], [25], [26], [27], [28] which incorporate variables such as clinical metrics, biomarker levels and family history of diabetes. The PreDx® Diabetes Risk Score (DRS) utilizes quantitative measures of seven blood-borne biomarkers (adiponectin, C-reactive protein, ferritin, glucose, hemoglobin A1C (HbA1c), interleukin 2 receptor α (IL2Ra) and insulin) in a multi-marker algorithm. DRS is a score from 1–10 which corresponds to the risk of developing T2DM within five years [21], [25]. The area under the receiver operating characteristic curve (AUC) of the DRS was 0.837 in a validation cohort obtained from a Danish population; this performance was significantly better compared to more common risk assessment tools such as HbA1c, fasting insulin, homeostasis model assessment of insulin resistance (HOMA-IR), fasting glucose or a noninvasive clinical model [25].

In this study the risk of developing T2DM was assessed using DRS or MetS in the Inter99 cohort, a group of adult Danish subjects who were followed for at least five years [29]. More specifically, the objective of this study was to compare the accuracy of each of these approaches, based on their use in current clinical practice, for identifying the subjects in this cohort who developed T2DM during the study using information obtained at baseline. The accuracy of these two prediction metrics for incident T2DM was assessed in an unselected population as well as a sub-population of the cohort which was considered to be at increased risk for diabetes based on the presence of IFG and/or elevated HbA1c (eHbA1c) [30].

## Materials and Methods

### Ethics Statement

The Inter99 study was approved by Copenhagen County Scientific Ethical Committee (reference number KA 98 155; ClinicalTrials.gov ID-no: NCT00289237) [29] and all participants gave written consent to participation in the study.

### Subjects

Subjects for this study were from the Inter99 cohort, a random population of subjects from the southwestern part of Copenhagen County, Denmark enrolled in a prevention study for cardiovascular disease. Although this was a lifestyle intervention trial for cardiovascular disease, the 5-year rate of progression to type 2 diabetes observed in this study (4.1%) was similar to other estimates of progression for this age group [31]. Of the 6,784 subjects who participated at baseline assessment, serum samples were available from 5,764 subjects. Subjects were excluded from the present study if they had T2DM at baseline, necessary measures needed for the analyses were not available, or assessment of diabetes outcomes was not available at five year follow-up. Diagnosis of T2DM was based upon criteria from the World Health Organization defined as either a 2-h plasma glucose of ≥11.1 mmol/L (≥200 mg/dL) from an oral glucose tolerance test (OGTT) or fasting plasma glucose (FPG) of ≥7.0 mmol/L (≥126 mg/dL) [32]. There were 4,128 subjects included in this study after applying the exclusion criteria. At each visit, data were collected regarding lifestyle, anthropometric measures (e.g. blood pressure, waist circumference, height, weight), routine laboratory measures (e.g. HbA1c, FPG and lipids), and OGTT as described previously [29].

### Determination of Diabetes Risk Score

The risk of developing T2DM within five years of baseline was expressed as DRS, which was calculated from quantitative measures of seven circulating biomarkers in baseline blood samples [21], [25]. Quantification of FPG, fasting serum insulin and HbA1c was determined at Steno Diabetes Center, Copenhagen, Denmark [29]. HbA1c concentrations were determined by ion exchange HPLC (Bio-Rad, USA) with a coefficient of variation of 11%. Quantification of fasting serum adiponectin, C-reactive protein, ferritin and IL2Ra was done at the Tethys Clinical Laboratory, Emeryville, CA. Ferritin was measured using solid-phase, two-site chemiluminescent immunometric assays. C-reactive protein (CRP) was measured using an immuno-turbidometric assay and adiponectin and IL2Ra were measured using a sandwich immunoassay format. In the validation of these assays, the coefficients of variation for ferritin, IL2Ra, CRP and adiponectin were 4.6%, 6.8%, 12.5% and 6.2%, respectively. DRS was previously trained on a nested case-control subset of 799 subjects from the Inter99 population [25]. For the current analysis, DRS was calculated from all available subjects (n = 4,128), including those from the case-control training study. Subjects with missing data for one or more DRS variables were excluded from the current analysis. All biomarker measurements in this study were obtained from previously unused aliquots and were run in a randomized order.

### Components of Metabolic Syndrome

Abnormal levels of components which are associated with the metabolic syndrome were: 1) elevated fasting glucose (≥5.6 mmol/L; 100 mg/dL); 2) elevated blood pressure (systolic ≥130 mmHg or diastolic ≥85 mmHg) or on anti-hypertensive drug treatment; 3) reduced fasting serum HDL cholesterol (<1.0 mmol/L/40 mg/dL in men or <1.3 mmol/L/50 mg/dL/ in women) or on lipid lowering drug treatment; 4) elevated fasting serum triglycerides (≥1.7 mmol/L/150 mg/dL) or on drug treatment; and 5) elevated waist circumference (≥80 cm in women; ≥94 cm in men) [12].

### Statistical Analysis

To evaluate the performance of DRS in assessing risk of developing diabetes, sensitivity and specificity of the DRS was compared to metabolic syndrome. Because the DRS is a continuous score, a DRS cut-point was selected to match the sensitivity of metabolic syndrome, a dichotomized variable, and specificity was then compared. Similarly, sensitivity was compared by selecting a second DRS cut-point to matching the specificity of metabolic syndrome. Statistical significance was evaluated by p-values obtained using 1000 bootstrap resamplings in which the entire procedure of selecting DRS cut-points and comparing sensitivity or specificity were embedded. The two-sided p-value was determined by the fraction of bootstrapped samples that resulted in an absolute difference of sensitivity (specificity) equal to or larger than the observed absolute difference on the entire data set. Additionally, multiple logistic regression analysis was done to combine DRS with metabolic syndrome in a model to determine if metabolic syndrome added predictive ability to DRS.

To further assess how DRS might impact clinical practice compared with MetS, Net Reclassification Improvement (NRI) was employed to compare the classification of subjects by DRS to the classification by number of MetS risk factors. This method of analysis was used in both the entire population as well as a sub-population considered at increased risk based on recent ADA Clinical Practice Guidelines that define individuals with; IFG or eHbA1c (i.e. 5.7%–6.4%) to be at increased risk for diabetes [30]. SSSubjects were categorized by the number of MetS factors present at baseline: 0–2, 3, or 4–5 factors. To enable reclassification analysis, two thresholds for the continuous DRS values were selected such that the population proportions in each of the three DRS categories (Group A, B and C) matched the proportions in the MetS risk-factor categories.

NRI was estimated as:
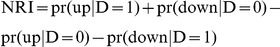
where D = 1 indicates conversion to diabetes, D = 0 indicates non-conversion, up indicates a higher risk classification by DRS than by MetS, and down indicates a lower classification. The statistical significance of the NRI was estimated by permutation testing with 10,000 random permutations of the outcome. The p-value was defined as the fraction of permutations that had greater NRI values than the observed NRI. A Binomial distribution was also used to find maximum likelihood estimates and confidence intervals for conversion to diabetic outcome. chi-square (χ2) test and Wilcoxon test were used to determine differences in factor and continuous variable, respectively. The statistical significance was accepted at the p = 0.05 level. Differences in mean of biomarker and MetS factors levels were determined by Analysis of Variance (ANOVA).

## Results

Table 1 describes the baseline clinical characteristics of the Inter99 population of 4,128 subjects for whom MetS risk factors also were available in this study. The overall 5-year conversion rate to T2DM was 4.1% (170 converters). All clinical, DRS biomarker variables and components of MetS were significantly different (p-value <0.05) between converters and non-converters with the exception of height.

**Table 1:** Baseline characteristics of subset of subjects from Inter99 cohort.

	Converters	Non-converters	P-value
Participants	170	3958	
Male sex	105 (61.8%)	1945 (49.1%)	0.0015
NFG	37 (21.8%)	2486 (62.8%)	0.0005
IFG	133 (78.2%)	1472 (37.2%)	0.0005
eHbA1c (%)	6.1 (5.8–6.4)	5.8 (5.5–6.0)	<0.0001
IFG or eHbA1c	160 (94.1%)	2909 (73.5%)	0.0005
Family history	55 (32.4%)	637 (16.1%)	0.0005
Age (years)	50.1 (45.0–55.0)	45.1 (40.0–50.2)	<0.0001
Height (cm)	172.0 (166.0–178.0)	172.0 (165.0–179.0)	0.8690
Weight (kg)	84.8 (75.3–98.0)	75.5 (66.0–86.3)	<0.0001
BMI (kg/m^2^)	28.6 (26.0–32.3)	25.3 (23.0–28.1)	<0.0001
Waist circumference (cm)	95 (87–107)	85 (76–94)	<0.0001
Hip circumference (cm)	105 (100–111)	100 (94–105)	<0.0001
Systolic blood pressure (mmHg)	140 (130–150)	128 (118–138)	<0.0001
Diastolic blood pressure (mmHg)	86 (80–95)	80 (75–90)	<0.0001
Fasting serum total cholesterol (mmol/L)	5.7 (5.0–6.5)	5.4 (4.7–6.1)	0.0002
Fasting serum HDL cholesterol (mmol/L)	1.2 (1.0–1.5)	1.4 (1.2–1.7)	<0.0001
Fasting serum LDL cholesterol (mmol/L)	3.5 (3.0–4.2)	3.4 (2.8–4.1)	0.0064
Fasting serum triglycerides (mmol/L)	1.6 (1.2–2.2)	1.0 (0.7–1.5)	<0.0001
Fasting serum insulin (pmol/L)	54.5 (35.0–78.0)	32.0 (23.0–48.0)	<0.0001
2-h serum insulin (pmol/L)	302.0 (187.0–448.0)	143.0 (88.0–227.0)	<0.0001
FPG (mmol/L)	6.1 (5.6–6.5)	5.4 (5.1–5.7)	<0.0001
2-h plasma glucose (mmol/L)	8.2 (6.9–9.4)	5.8 (4.9–6.7)	<0.0001
Fasting serum adiponectin (µg/mL)	6.7 (5.4–8.5)	8.4 (6.6–10.8)	<0.0001
Fasting serum CRP (mg/L)	2.2 (1.0–4.0)	0.9 (0.4–2.4)	<0.0001
Fasting serum ferritin (ng/mL)	150.0 (70.3–289.0)	90.7 (41.3–173.0)	<0.0001
Fasting serum IL-2Rα (U/mL)	386.8 (308.4–487.8)	356.3 (285.5–446.7)	0.0026

Data are *n* (%) or median (interquartile range) for continuous variables. For categorical descriptors, values are counts (percentage of total for that cohort). Differences in frequency between converters and nonconverters were evaluated with a Monte Carlo estimation of the χ^2^ statistic (2,000 replicates). Differences in medians of continuous variables were evaluated with a Wilcoxon test. NFG, normal fasting glucose; IFG, impaired fasting glucose; eHbA1c, elevated hemoglobin HbA1c.

### Performance of MetS and DRS in Unselected Population

The accuracy of MetS and DRS, as used in clinical practice, for assessing risk of developing diabetes is compared in Table 2. Since in clinical practice, MetS is used as a dichotomous variable, we used MetS in this way in our analysis. The sensitivity of metabolic syndrome to predict incident diabetes was 71.4% with a false positive rate of 27.7%. When DRS was matched on false positive rate to metabolic syndrome, DRS had a significantly higher sensitivity (79.9%, p value = 0.023). Similarly, when DRS was matched on sensitivity (71.4%) to metabolic syndrome, DRS has a significantly lower false positive rate (20.0%, p value = 0.011). When metabolic syndrome was used in combination with DRS, there was no significant change in the AUC. The ATP-III thresholds for waist circumference (88 cm for women and 102 cm for men) were also employed in the analysis (Third Report [33]), and, while there was an overall decrease in sensitivity and false-positive rate, DRS was significantly better than MetS by both criteria (p value <0.05).

**Table 2:** Comparison of risk assessment of Metabolic Syndrome and DRS.

	AUC	Sensitivity (%)	False-positive rate (%)	*P-value and **CI	#PPV	∧NPV
Metabolic Syndrome	-	71.4	27.7			
Diabetes Risk Score (DRS)	0.836	79.9	27.7 (fixed)	0.023 (0.022–0.157)	10.0%	98.3%
DRS plus Metabolic Syndrome	0.840 (P = 0.128)	79.2	27.7 (fixed)	0.021 (0.018–0.144)		
Metabolic Syndrome	-	71.4	27.7			
DRS		71.4 (fixed)	20.0	0.011 (0.019–0.139)	13.2%	98.5%
DRS plus Metabolic Syndrome		71.4 (fixed)	17.4	0.009 (0.038–0.0150)		

*P for comparison with the row immediately above.

**95% C.I based on observed variance under bootstrap resampling of the differences to MetS.

# = Positive Predictive Value.

∧ = Negative Predictive Value.

Reclassification of subjects by DRS in the three Met S groups (i.e. subjects with 0–2, 3, or 4–5 risk factors at baseline) is summarized in Table 3. Cut-off scores for DRS were established such that the study cohort was parsed into three groups, each with the same proportion of subjects as each of the three MetS groups (DRS Group A, <4.4; DRS Group B, 4.4–6.8; DRS Group C, >6.8). The numbers of subjects, population percent, observed rate of conversion to diabetes during the five year follow-up and the total number of subjects for each DRS group or MetS group are shown for each combination of MetS and DRS categories. The number of subjects reclassified by DRS for each MetS category also is shown (Reclassified Subtotal).

**Table 3:** Reclassification of all subjects in three MetS groups by DRS.

# MetS Risk Factors	Variable	DRS Score			Subjects	
		A	B	C	Total	Reclassified Subtotal
		< = 4.4	4.4–6.8	>6.8		
0–2 Factors	No. of Obs.	2457	362	93	2912	455
	% of Pop.	59.5%	8.8%	2.3%	70.5%	15.6%
	(95% C.I.)	(58.0%–61.0%)	(7.9%–9.7%)	(1.8%–2.8%)	(69.1%–71.9%)	(14.3%–17.0%)
	Conv. Rate	1.1%	3.0%	12.9%	1.7%	
	(95% C.I.)	(0.7%–1.5%)	(1.5%–5.4%)	(6.8%–21.5%)	(1.2%–2.2%)	
3 Factors	No. of Obs.	333	247	166	746	499
	% of Pop.	8.1%	6.0%	4.0%	18.1%	66.9%
	(95% C.I.)	(7.3%–8.9%)	(5.3%–6.8%)	(3.4%–4.7%)	(16.9%–19.3%)	(63.4%–70.3%)
	Conv. Rate	2.7%	6.1%	18.7%	7.4%	
	(95% C.I.)	(1.2%–5.1%)	(3.4%–9.8%)	(13.1%–25.4%)	(5.6%–9.5%)	
4–5 Factors	No. of Obs.	122	137	211	470	259
	% of Pop.	3.0%	3.3%	5.1%	11.4%	55.1%
	(95% C.I.)	(2.5%–3.5%)	(2.8%–3.9%)	(4.5%–5.8%)	(10.4%–12.4%)	(50.5%–59.7%)
	Conv. Rate	0.0%	14.6%	21.8%	14.0%	
	(95% C.I.)	(0.0%–3.0%)	(9.2%–21.6%)	(16.4%–28%)	(11.0%–17.5%)	
**Total**	No. of Obs.	2912	746	470	4128	1213
	% of Pop.	70.5%	18.1%	11.4%	100.0%	29.4%
	(95% C.I.)	(69.1%–71.9%)	(16.9%–19.3%)	(10.4%–12.4%)	(99.9%–100.0%)	(28.0%–30.8%)
	Conv. Rate	1.2%	6.2%	18.9%	4.1%	
	(95% C.I.)	(0.8%–1.7%)	(4.5%–8.1%)	(15.5%–22.8%)	(3.5%–4.8%)	
**NRI, (P-value)**	0.146 (0.0008)					
**NRI, Converters**	0.147					
**NRI, Nonconverters**	−0.001					

The overall NRI of 0.146 (p = 0.0008) shows that DRS significantly improved the classification of subjects at risk for diabetes compared to risk assessment by the number of MetS factors. Additionally, the reclassification of converters accounts for most of the NRI (0.147) with a modest reclassification of non-converters (NRI = −0.001), demonstrating an increased specificity of DRS over MetS without a reduction in sensitivity. The largest proportion of subjects reclassified by DRS was in the group of subjects with 3 MetS risk factors. The observed conversion rate to T2DM for the 746 subjects in the MetS group with 3 risk factors was 7.4%. DRS reclassified 499 (66.9%) of those subjects; 166 subjects were classified in DRS Group C (i.e. highest risk) with an observed an observed conversion rate of 18.7% and 333 subjects were classified into DRS Group A (lowest risk) with an observed conversion rate of 2.7%.

Among the 470 subjects in the group of 4–5 MetS risk factors, none of the 122 subjects reclassified by DRS into group A (lowest risk) converted to diabetes within five years despite having an elevated risk as assessed by MetS. An ANOVA analysis was performed for each biomarker quantified for determination of the DRS and for each MetS risk factor to identify differences in this cohort with 4–5 MetS factors (Table 4). All biomarkers were significantly different between the three DRS groups (p<0.001 for each marker). Among the MetS risk factors, only mean fasting plasma glucose, waist, and systolic blood pressure were significantly different (p-values <0.001, 0.003 and 0.03 respectively) between the three DRS groups and increased with increasing DRS. However, triglycerides and HDL were not significantly different between the three DRS groups. Further supporting the accuracy of DRS in assessing risk is the observation that the 93 subjects in the 0–2 MetS group who were reclassified in highest risk by DRS (Group C) had a higher conversion rate than those subjects reclassified to low risk by DRS (Group A) in the 4–5 MetS category (12.9% vs. 0%, respectively).

**Table 4:** Mean biomarker and risk factor values by DRS for subjects with 4–5 MetS factors.

Variables	Mean (SD)			P-value
	A	B	C	
Fasting serum adiponectin (µg/mL)	7.85 (2.3)	7.05 (2.3)	5.95 (2.0)	<0.001
Fasting serum CRP (mg/L)	1.98 (2.4)	3.14 (3.3.)	5.05 (7.3)	<0.001
Fasting serum ferritin (ng/mL)	132.09 (178.6)	177.54 (166.7)	237.32 (205.4)	<0.001
Fasting serum IL-2Rα (U/mL)	368.65 (156.1)	397.93 (124.3)	450.59 (155.5)	<0.001
Fasting serum insulin (pmol/L)	55.11(26.9)	60.18 (30.1)	70.48 (33.9)	<0.001
HbA1c (%)	5.76 (0.3)	5.89 (0.4)	6.06 (0.5)	<0.001
FPG (mmol/L)	5.54(0.4)	5.81 (0.4)	6.17 (0.4)	<0.001
Fasting serum triglycerides (mmol/L)	2.32 (1.4)	2.4 (1.2)	2.63 (2.0)	0.182
Fasting serum HDL cholesterol (mmol/L)	1.03 (0.2)	1.06 (0.2)	1.06 (0.2)	0.471
Waist circumference (cm)	97 (8)	99 (10)	101 (12)	0.003
Systolic blood pressure (mmHg)	137 (13)	139 (13)	141 (14)	0.029
Diastolic blood pressure (mmHg)	88 (8)	89 (9)	89 (10)	0.452

Mean levels of biomarker and MetS variables within each DRS group (A–C) for all subjects with 4–5 MetS risk factors (n = 470). The P-values are calculated by ANOVA.

### Performance of MetS and DRS in Population at Increased Risk for T2DM

The reclassification of subjects with IFG (fasting plasma glucose between 5.6 and 6.9 mmol/L) and/or eHbA1c (HbA1c between 5.7%–6.4%), a sub-population considered to be at increased risk for T2DM is summarized in Table 5. Subjects with IFG had a mean HbA1c of 5.9% with a range of 4.0–7.6%. Cut-off scores for DRS were determined such that this sub-population was parsed into three groups, each with the same proportion of subjects as each of the three MetS groups (DRS Group A, <4.6; DRS Group B,4.6–6.9; DRS Group C, >6.9) as done previously in Table 3. Approximately 74% of subjects (3,069 of 4,128 subjects) in this study met one or both of these criteria for increased risk. A shift of subjects to higher risk groups for T2DM was observed based on risk assessment by MetS or DRS. Patterns of reclassification by DRS which were similar to those observed in the total population were observed in this population at increased risk. As observed with the total population, the largest number of subjects reclassified by DRS was in the 3 MetS factor group. Subjects in the MetS group with 4–5 risk factors which were reclassified to DRS Group A had a conversion rate of 0%, as observed in the total population. The relative risk between high and low DRS groups (1.5%–19.7%, relative risk 13.1) was 1.8 fold greater than the relative risk between the high and low MetS groups (2.1%–15.0%, relative risk 7.1), consistent with the results observed in the total population. There is a significant NRI of 0.141 with a p-value of 0.0032, further highlighting the improved risk stratification of DRS compared to MetS even in an increased risk population. As in the total population, the NRI is dominated by the correct reclassification of converters with a modest reclassification of non-converters (NRI = 0.144 and −0.003, respectively).

**Table 5:** Reclassification of subjects with impaired fasting glucose and/or elevated HbA1c in three MetS groups by DRS.

# MetS Risk Factors	Variable	DRS Score			Subjects	
		A	B	C	Total	Reclassified Subtotal
		< = 4.6	4.6–6.9	>6.9		
1–2 Factors	No. of Obs.	1556	318	85	1959	403
	% of Pop.	50.7%	10.4%	2.8%	63.8%	20.6%
	(95% C.I.)	(48.9%–52.5%)	(9.3%–11.5%)	(2.2%–3.4%)	(62.1%–65.5%)	(18.8%–22.4%)
	Conv. Rate	1.3%	3.0%	12.9%	2.1%	
	(95% C.I.)	(0.8%–2.0%)	(1.5%–5.7%)	(6.6%–22.0%)	(1.5%–2.8%)	
3 Factors	No. of Obs.	286	218	159	663	445
	% of Pop.	9.3%	7.0%	5.0%	21.6%	67.1%
	(95% C.I.)	(8.3%–10.4%)	(6.2%–8.1%)	(4.4%–6.0%)	(20.2%–23.1%)	(63.4%–70.7%)
	Conv. Rate	3.1%	6.0%	19.5%	8.0%	
	(95% C.I.)	(1.4%–5.9%)	(3.2%–10.0%)	(13.6%–26.5%)	(6.0%–10.3%)	
4–5 Factors	No. of Obs.	117	127	203	447	244
	% of Pop.	4.0%	4.1%	6.6%	14.6%	54.6%
	(95% C.I.)	(3.2%–4.6%)	(3.5%–4.9%)	(5.8%–7.6%)	(13.3%–15.9%)	(49.8%–59.3%)
	Conv. Rate	0.0%	15.7%	22.7%	15.0%	
	(95% C.I.)	(0.0%–3.1%)	(9.9%–23.3%)	(17.1%–29.0%)	(11.6%–18.4%)	
**Total**	No. of Obs.	1959	663	447	3069	1092
	% of Pop.	63.8%	21.6%	14.6%	100.0%	35.6%
	(95% C.I.)	(62.1%–65.5%)	(20.2%–23.1%)	(13.3%–15.9%)	(99.9%–100.0%)	(33.9%–37.3%)
	Conv. Rate	1.5%	6.5%	19.7%	5.2%	
	(95% C.I.)	(1.0%–2.1%)	(4.7%–8.6%)	(16.1%–23.7%)	(4.5%–6.1%)	
**NRI (P-value)**	0.1407 (0.0032)					
**NRI, Converters**	0.1438					
**NRI, Nonconverters**	−0.0031					

## Discussion

The objective of this study was to compare the performance of two risk assessment tools, MetS and DRS, in assessing subjects' risk of developing T2DM. The current analysis shows that DRS provides a superior assessment of diabetes risk compared to MetS by allowing for more accurate risk stratification of subjects in this population. Additionally, a model combining MetS with DRS did not improve the AUC demonstrating that metabolic syndrome does not provide additional predictive information to DRS.

The absolute risk of diabetes during 5 years in this population was 4.1% (Table 3), similar to the risk observed in the United States [31]. Using MetS alone to assess diabetes risk has a sensitivity of 71.4% and a false positive rate of 27.7% (Table 2). While presence of MetS is sensitive to identify those subjects at risk for developing diabetes, it lacks specificity. In this study, about 30% of subjects were classified as having MetS. Among subjects with MetS the conversion rate was 10.0% while those without MetS (2 or fewer factors) had a conversion rate of 1.7%, a 5.8 fold difference in risk, indicating that the presence or absence of MetS provides a basic level of risk stratification in the total population (Table 3). However, subjects in the highest risk DRS group (Group C) had a conversion rate of 18.9% compared to a conversion rate of 2.2% for subjects in the lower risk DRS groups (Group A and B).

Counting the number of factors of the MetS present in subjects provided further stratification of risk for T2DM; subjects with three risk factors had a conversion rate of 7.4% while those with 4–5 factors had a conversion rate of 14.0%. However, among those same subjects with 3 MetS factors, DRS was able to further stratify subjects with a higher degree of accuracy as well as a wider range of stratification in both the total population and the population at increased risk (Table 3 and 5). This increased accuracy of DRS in reclassifying subjects with 3 MetS risk factors to either lower or higher risk of diabetes provides potential clinical value by allowing intervention to be focused on those individuals at highest risk in this group who might be considered to be at intermediate risk by risk factor counting.

Among those subjects with 4–5 MetS factors, 122 were reclassified as lowest risk by DRS (Group A, Table 3). In fact, no subjects in DRS Group A converted to diabetes despite being classified at highest risk by MetS. As shown in Table 4 all biomarkers were significantly different between DRS groups (p<0.001) while only three MetS factors, fasting glucose, waist and systolic blood pressure showed significant, although small, differences between these DRS groups. The improved risk assessment provided by DRS is a function of changes in the seven markers that are representative of the multiple pathways that are dysregulated in the development of diabetes.

Overall, DRS provided an enhancement of risk stratification in comparison with counting the number of MetS risk factors. Within the group of subjects with 0–2 MetS risk factors those in Group C by DRS (highest risk) had a higher conversion rate than those subjects reclassified to Group A (lowest risk) by DRS in the 4–5 MetS category (12.9% vs. 0%, respectively, Table 3). The stratification of risk was greater using DRS than the number of MetS factors when looking at the highest and lowest risk groups. Subjects in DRS Group C (highest risk conversion rate = 18.9%) had 15-fold higher rate of conversion than did subjects in DRS Group A (lowest risk, conversion rate = 1.2%). By comparison, there was only an 8-fold difference in conversion rates between the lowest MetS risk group (i.e. 0–2 factors; 1.7% conversions) and the highest MetS risk group (i.e. 4–5 factors; 14.0% conversion) (Table 3).

A large proportion of the population was identified in the lowest risk category by both MetS and DRS (59.5% of total subjects) with a conversion rate of 1.1%. However, it is important to note that all subjects classified in the lowest risk category by DRS (Group A) had a lower risk of conversion to T2DM regardless of the number of MetS factors in both the total and at risk populations (Tables 3 and 5).

In this study, two of the three ADA defined risk factors for T2DM (IFG and eHbA1c) (28) were used to identify a sub-population at increased risk. IFG and eHbA1c were chosen because they can easily be measured in routine clinical practice. The results show that DRS more accurately stratified the risk of T2DM in subjects in this increased risk sub-population (Table 5). This highlights the improved performance provided by the combination of biomarkers in DRS compared to MetS factors in accurately identifying those subjects at increased risk for T2DM even within the population already identified to be at increased risk.

Both the economic burden and impact on quality of life that result once an individual becomes diabetic are dramatic [3]. Several long-term prospective clinical trials have shown that lifestyle intervention can delay and possibly prevent conversion to T2DM in high-risk individuals [4], [5], confirming the need for easily accessible clinical tests that accurately identify those at highest risk of conversion. With improved risk stratification methods, clinicians could focus limited resources and intervention strategies on those individuals most in need. Although OGTT has been identified as a strong predictor of diabetes risk, it is rarely used in clinical practice due to level of difficulty in administering the test and lack of reproducibility of results [34]. IFG has been shown to lack specificity in identifying those at highest risk [6], [7], [8], [9], [10]. MetS factors are easily assessable measures for clinicians but their utility in assessing diabetes risk has been widely debated [11], [13], [14], [35], [36]. As previously noted, Met Syndrome is relatively sensitive; it has very limited clinical relevance due to the low specificity. A recent report based on the evaluation of National Health and Nutrition Examination Survey (NHANES) 2003–2006 estimated that over one third of the US adult population had MetS [37] and thus would be identified as being at risk for developing diabetes. By comparison, DRS is a simple laboratory test available to clinicians and it may reflect metabolic disturbances in pathways involved with diabetes better than MetS factors. DRS provides improved specificity compared to MetS. This improved specificity was shown here to provide improved risk stratification and accuracy in assessing risk of T2DM compared to MetS.

Several limitations are noted. One limitation of this study is that the DRS algorithm was originally trained on a subset of the Inter 99 population, potentially biasing the results presented here. However, the training population was a relatively small portion of the subjects, which were used in the present study and only a fraction of the available converters and non-converters present were employed in the training of DRS, thus limiting the potential bias. Additionally, the performance observed in the sequestered validation samples was equivalent to the performance in the full cohort used in the current study as measured by the Area Under the ROC Curve (0.838 and 0.837, respectively) (24), minimizing any indication of bias in the results presented here.

Given that there are no guidelines for selecting thresholds for DRS risk groups, two approaches for the reclassification analysis were possible. DRS thresholds for assignment to risk groups could be done by matching risk across categories or by matching population proportions across categories. We selected the latter approach. By pre-specifying DRS thresholds based on matching population proportions potential bias was minimized.

In summary, given the increasing rates of diabetes worldwide, better tools to identify subjects at highest risk of developing diabetes are needed. In this study the performance of the multi-marker DRS model was compared to presence of MetS factors for assessing risk of incident T2DM. DRS provided improved risk stratification in all MetS risk factor groups. DRS provides physicians with a tool to better identify subjects at increased risk for developing T2DM and allows for interventions to be targeted to those subjects at highest risk.
